# Supplementary material for: APOL1 renal risk variants have contrasting resistance and susceptibility associations with African trypanosomiasis
Source: eLife. 2017 May 24;6:e25461. doi: 10.7554/eLife.25461 (PMC5495568; doi:10.7554/eLife.25461)
Supplement: Figure 2—source data 1. — Where data for G1 allele frequency were unavailable, data are inferred from rs73885319 only. N/A: Data not available, CEPH-HGDP: Centre d’Etude du Polymorphisme Humain - Human Genome Diversity Panel, TCGA-UCL: The Centre for Genetic Anthropology at University College London. DOI: http://dx.doi.org/10.7554/eLife.25461.017 [file elife-25461-fig2-data1.docx]

| Country | Population/  sampling location | Latitude | Longitude | *N* | Allele Frequency | | Reference |
| --- | --- | --- | --- | --- | --- | --- | --- |
|  |  |  |  |  | G1  (rs73885319 + rs60910145)  % | G2  rs71785313  % |  |
| Algeria | Mozabite (HGDP-CEPH) | 32.0 | 3.0 | 30 | 1.8 | 0 | Kopp *et al*.^1^ |
| Angola | Himbe | -17.0 | 12.6 | 67 | 14.9 | 18.7 | *Pinto et al.*^2^ |
| Angola | Kuvale | -15.6  -14.6 | 13.1  13.2 | 78 | 4.5 | 9.6 | *Pinto et al.*^2^ |
| Angola | Kwepe | -15.8 | 12.1 | 33 | 3.0 | 10.6 | *Pinto et al.*^2^ |
| Angola | Kwisi | -15.9 | 12.6 | 21 | 0.0 | 14.3 | *Pinto et al.*^2^ |
| Angola | !Xuun | -16.3 | 16.0 | 35 | 0.0 | 1.4 | *Pinto et al.*^2^ |
| Botswana | Motswana-Gaberone | -24.6 | 25.9 | 570 | 5.5 | 5.5 | Limou et al.^3^ |
| Cameroon | Far-North-CMR/Chad  (TCGA-UCL) | 12.5 | 14.5 | 64 | 0.8 | 3.3 | Tzur *et al*.^4^  Behar *et al*.^5^ |
| Cameroon | Somie  (TCGA-UCL) | 6.5 | 11.5 | 65 | 16.4 | 12.3 | Tzur *et al*.^4^  Behar *et al*.^5^ |
| Central African Republic | Biaka Pygmy  (HGDP-CEPH) | 4.0 | 17.0 | 36 | 4.2 | 8.3 | Kopp *et al*.^1^ |
| Ethiopia | Afar  (TCGA-UCL) | 12.0 | 41.5 | 76 | 0.0 | 0.0 | Tzur *et al*.^4^  Behar *et al*.^5^ |
| Ethiopia | Amhara  (TCGA-UCL) | 11.5 | 38.5 | 76 | 0.0 | 0.0 | Tzur *et al*.^4^  Behar *et al*.^5^ |
| Ethiopia | Annuak  (TCGA-UCL) | 8.3 | 34.6 | 76 | 2.0 | 2.7 | Tzur *et al*.^4^  Behar *et al*.^5^ |
| Ethiopia | Maale  (TCGA-UCL) | 7.6 | 37.2 | 76 | 0.0 | 0.0 | Tzur *et al*.^6^  Behar *et al*.^5^ |
| Ethiopia | Oromo  (TCGA-UCL) | 9 | 38.7 | 76 | 0.0 | 0.0 | Tzur *et al*.^4^  Behar *et al*.^5^ |
| Ghana | Akan | 6.7 | -1.6 | 171 | 43.0 | 11.0 | Thomson *et al*.^7^ |
| Ghana | Asante  (TCGA-UCL) | 5.8 | -2.8 | 35 | 40.9 | 12.9 | Tzur *et al*.^4^  Behar *et al*.^5^ |
| Ghana | Bulsa  (TCGA-UCL) | 10.7 | -1.3 | 22 | 11.4 | 21.4 | Tzur *et al*.^4^  Behar *et al*.^5^ |
| Ghana | Ga-Adangbe | 5.6 | -0.2 | 139 | 27.0 | 17.0 | Thomson *et al*.^7^ |
| Guinea | Coastal mangrove area | 9.8 | -13.6 | 104 | 15.4 | 18.8 | This study |
| Kenya | Kikuyu | -0.4 | 37.0 | 112 | 5.0 | 6.0 | Thomson *et al*.^7^ |
| Kenya | Luo | -0.5 | 34.7 | 895 | 8.0 | 9.0 | Thomson *et al*.^7^ |
| Kenya | Luyha in Webuye (HAPMAP) | 0.6 | 34.6 | 90 | 5.0 | 7.0 | Kopp *et al*.^1^ |
| Kenya | Masai | -1.1 | 35.9 | 102 | 2.0 | 3.0 | Thomson *et al*.^7^ |
| Malawi | MWI  (TCGA-UCL) | -13.9 | 33.7 | 50 | 12.0 | 12.0 | Tzur *et al*.^4^  Behar *et al*.^5^ |
| Mozambique | Sena  (TCGA-UCL) | -17.5 | 35.0 | 51 | 12.2 | 11.0 | Tzur *et al*.^4^  Behar *et al*.^5^ |
| Mozambique | Shangaan | -24.3 | 32.9 | 23 | 17.4 | 21.7 | *Pinto et al.*^2^ |
| Nigeria | Esan | 9.1 | 7.4 | 99 | 49.0 | N/A | 1000 genomes project^8^ |
| Nigeria | Ibo | 6.5 | 7.5 | 190 | 49.0 | 17.0 | Thomson *et al*.^7^ |
| Nigeria | Yoruba  (HapMap) | 7.4 | 3.9 | 180 | 38.0 | 8.0 | Genovese *et al*.^9^ |
| Republic of the Congo | COG/Bakongo (TCGA-UCL) | -4.3 | 15.3 | 55 | 10.9 | 4.5 | Tzur *et al*.^4^  Behar *et al*.^5^ |
| São Tomé e Príncipe | Príncipe | 0.25 | 6.6 | 153 | 20.3 | 10.1 | *Pinto et al.*^2^ |
| Senegal | Mandenka  (HGDP-CEPH) | 12.0 | -12.0 | 24 | 5.0 | 20.0 | Kopp *et al*.^1^ |
| Sierra Leone | Mende | 8.6 | -11.8 | 85 | 12.0 | N/A | 1000 genomes project^8^ |
| Somalia | Somali | 2.0 | 45.4 | 30 | 0.0 | 2.0 | Thomson *et al*.^7^ |
| South Africa | Capetown –  mixed ancestry | -33.9 | 18.4 | 859 | 3.6 | 5.8 | Matsha *et al*.^10^ |
| South Africa | Zulu-Durban | -29.9 | 30.9 | 113 | 5.3 | 5.5 | Limou et al.^3^ |
| Sudan | Kordofan  (TCGA-UCL) | 13.1 | 30.4 | 30 | 0.0 | 5.0 | Tzur *et al*.^4^  Behar *et al*.^5^ |
| The Gambia | Western division-Mandinka | 13.2 | -16.3 | 116 | 24.0 | N/A | 1000 genomes project^8^ |
| Uganda | Soroti/  Kaberamaido | 1.9 | 33.3 | 180 | 3.3 | 7.2 | This study |

**References**

1. Kopp, J.B., Nelson, G.W., Sampath, K., Johnson, R.C., Genovese, G., An, P., Friedman, D., Briggs, W., Dart, R., Korbet, S., et al. (2011). APOL1 genetic variants in focal segmental glomerulosclerosis and HIV-associated nephropathy. J. Am. Soc. Nephrol. *22*, 2129–2137.

2. Pinto, J.C., Oliveira, S., teixeira, S., Martins, D., Fehn, A.-M., Aço, T., Gayà-Vidal, M., and Rocha, J. (2016). Food and pathogen adaptations in the Angolan Namib desert: Tracing the spread of lactase persistence and human African trypanosomiasis resistance into southwestern Africa. Am. J. Phys. Anthropol.

3. Limou, S., Nelson, G.W., Kopp, J.B., and Winkler, C.A. (2014). APOL1 kidney risk alleles: population genetics and disease associations. Adv Chronic Kidney Dis *21*, 426–433.

4. Tzur, S., Rosset, S., Shemer, R., Yudkovsky, G., Selig, S., Tarekegn, A., Bekele, E., Bradman, N., Wasser, W.G., Behar, D.M., et al. (2010). Missense mutations in the APOL1 gene are highly associated with end stage kidney disease risk previously attributed to the MYH9 gene. Hum. Genet. *128*, 345–350.

5. Behar, D.M., Kedem, E., Rosset, S., Haileselassie, Y., Tzur, S., Kra-Oz, Z., Wasser, W.G., Shenhar, Y., Shahar, E., Hassoun, G., et al. (2011). Absence of APOL1 risk variants protects against HIV-associated nephropathy in the Ethiopian population. Am. J. Nephrol. *34*, 452–459.

6. Monajemi, H., Fontijn, R.D., Pannekoek, H., and Horrevoets, A.J.G. (2002). The apolipoprotein L gene cluster has emerged recently in evolution and is expressed in human vascular tissue. Genomics *79*, 539–546.

7. Thomson, R., Genovese, G., Canon, C., Kovacsics, D., Higgins, M.K., Carrington, M., Winkler, C.A., Kopp, J., Rotimi, C., Adeyemo, A., et al. (2014). Evolution of the primate trypanolytic factor APOL1. Proc. Natl. Acad. Sci. U.S.a. *111*, E2130–E2139.

8. 1000 Genomes Project Consortium, Abecasis, G.R., Auton, A., Brooks, L.D., DePristo, M.A., Durbin, R.M., Handsaker, R.E., Kang, H.M., Marth, G.T., and McVean, G.A. (2012). An integrated map of genetic variation from 1,092 human genomes. Nature *491*, 56–65.

9. Genovese, G., Friedman, D.J., Ross, M.D., Lecordier, L., Uzureau, P., Freedman, B.I., Bowden, D.W., Langefeld, C.D., Oleksyk, T.K., Knob, A.U., et al. (2010). Association of Trypanolytic ApoL1 Variants with Kidney Disease in African-Americans. Science *329*, –845.

10. Matsha, T.E., Kengne, A.P., Masconi, K.L., Yako, Y.Y., and Erasmus, R.T. (2015). APOL1 genetic variants, chronic kidney diseases and hypertension in mixed ancestry South Africans. BMC Genet. *16*, 69.
